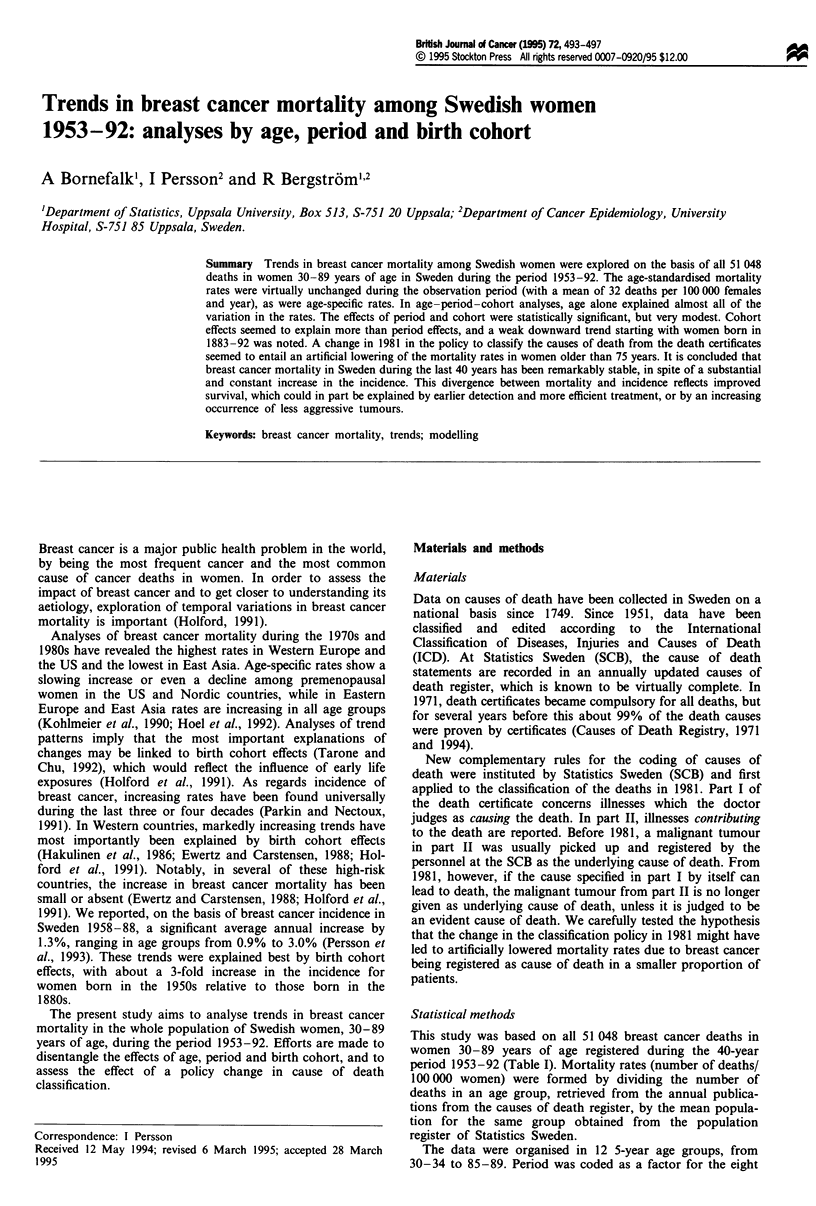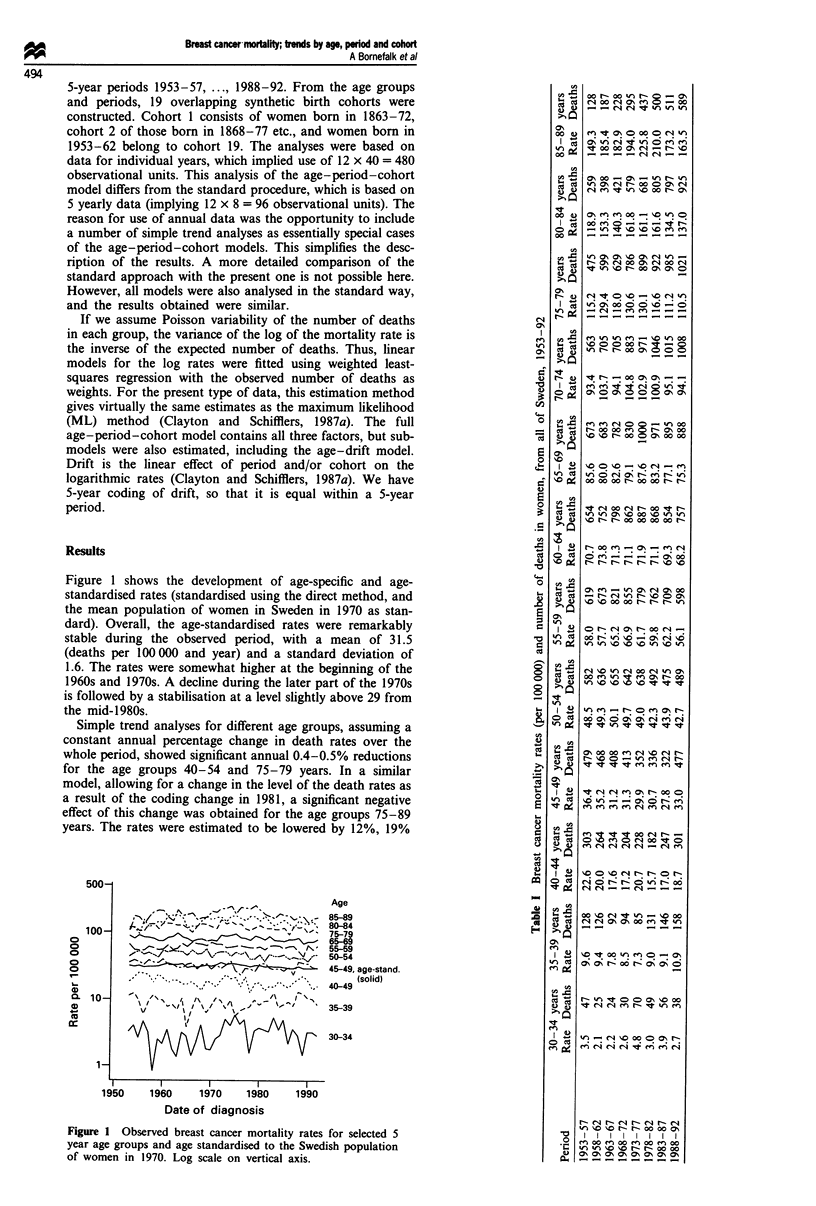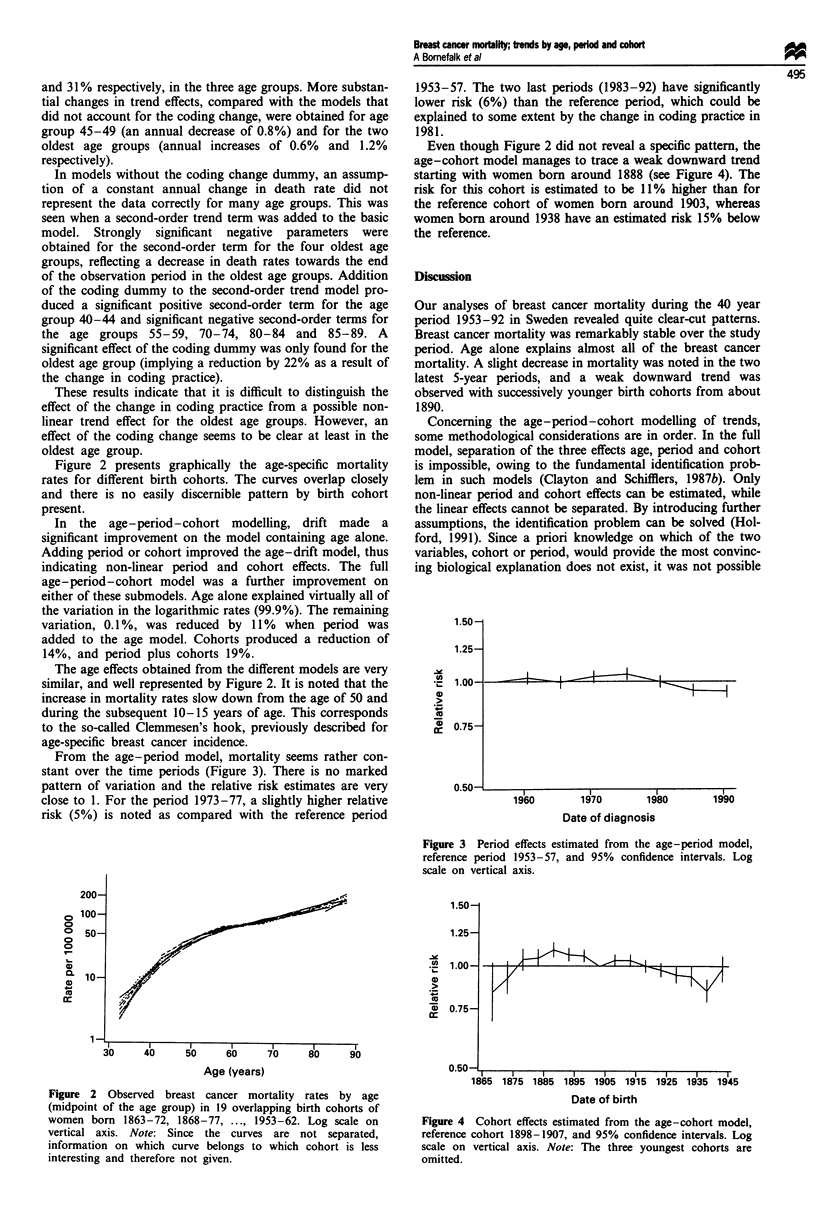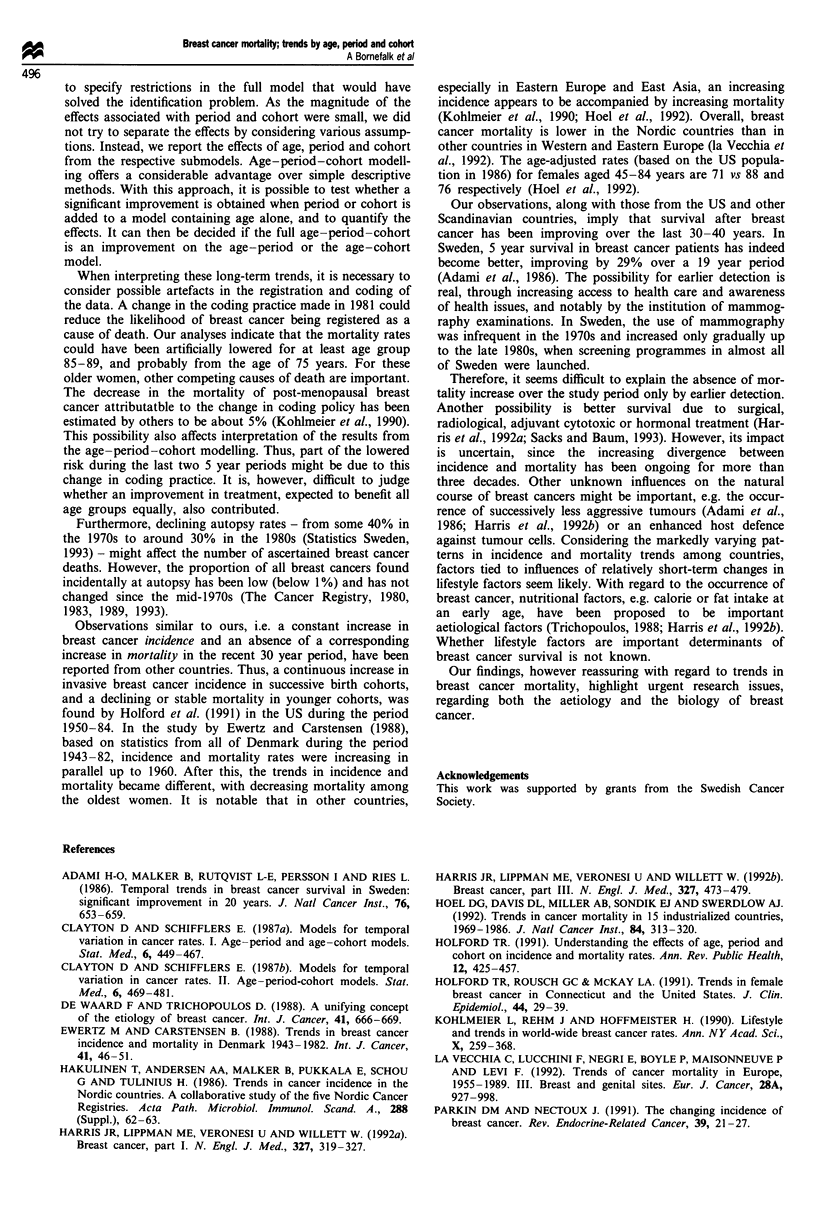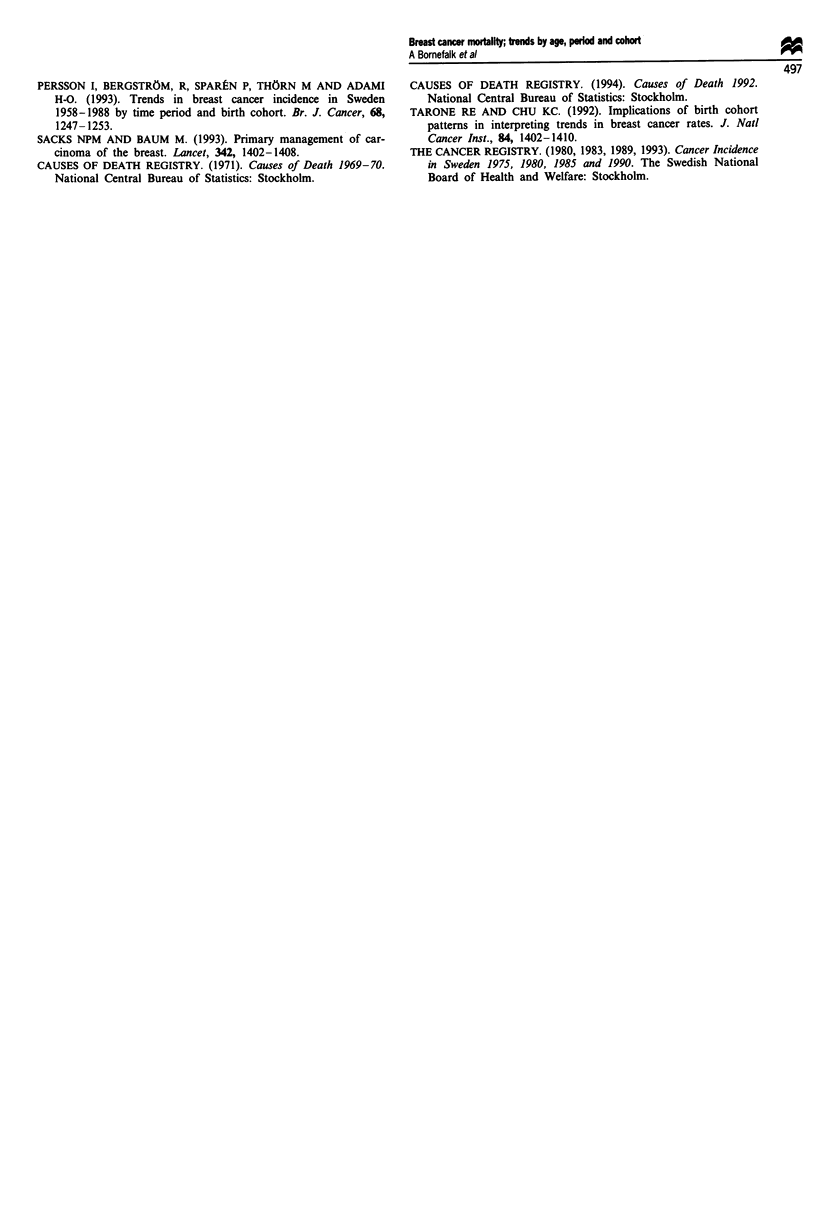# Trends in breast cancer mortality among Swedish women 1953-92: analyses by age, period and birth cohort.

**DOI:** 10.1038/bjc.1995.361

**Published:** 1995-08

**Authors:** A. Bornefalk, I. Persson, R. Bergström

**Affiliations:** Department of Statistics, Uppsala University, Sweden.

## Abstract

Trends in breast cancer mortality among Swedish women were explored on the basis of all 51,048 deaths in women 30-89 years of age in Sweden during the period 1953-92. The age-standardised mortality rates were virtually unchanged during the observation period (with a mean of 32 deaths per 100,000 females and year), as were age-specific rates. In age-period-cohort analyses, age alone explained almost all of the variation in the rates. The effects of period and cohort were statistically significant, but very modest. Cohort effects seemed to explain more than period effects, and a weak downward trend starting with women born in 1883-92 was noted. A change in 1981 in the policy to classify the causes of death from the death certificates seemed to entail an artificial lowering of the mortality rates in women older than 75 years. It is concluded that breast cancer mortality in Sweden during the last 40 years has been remarkably stable, in spite of a substantial and constant increase in the incidence. This divergence between mortality and incidence reflects improved survival, which could in part be explained by earlier detection and more efficient treatment, or by an increasing occurrence of less aggressive tumours.


British Journal of Cancer (1995) 72, 493-497
© 1995 Stockton Press All rights reserved 0007-0920/95 $12.00

### Trends in breast cancer mortality among Swedish women 1953-92: analyses by age, period and birth cohort

A Bornefalk[1], I Persson[2] and R Bergström[1,2]

[1]Department of Statistics, Uppsala University, Box 513, S-751 20 Uppsala; [2]Department of Cancer Epidemiology, University Hospital, S-751 85 Uppsala, Sweden.

**Summary** Trends in breast cancer mortality among Swedish women were explored on the basis of all 51 048 deaths in women 30-89 years of age in Sweden during the period 1953-92. The age-standardised mortality rates were virtually unchanged during the observation period (with a mean of 32 deaths per 100 000 females and year), as were age-specific rates. In age-period-cohort analyses, age alone explained almost all of the variation in the rates. The effects of period and cohort were statistically significant, but very modest. Cohort effects seemed to explain more than period effects, and a weak downward trend starting with women born in 1883-92 was noted. A change in 1981 in the policy to classify the causes of death from the death certificates seemed to entail an artificial lowering of the mortality rates in women older than 75 years. It is concluded that breast cancer mortality in Sweden during the last 40 years has been remarkably stable, in spite of a substantial and constant increase in the incidence. This divergence between mortality and incidence reflects improved survival, which could in part be explained by earlier detection and more efficient treatment, or by an increasing occurrence of less aggressive tumours.

**Keywords:** breast cancer mortality, trends; modelling

Breast cancer is a major public health problem in the world, by being the most frequent cancer and the most common cause of cancer deaths in women. In order to assess the impact of breast cancer and to get closer to understanding its aetiology, exploration of temporal variations in breast cancer mortality is important (Holford, 1991).

Analyses of breast cancer mortality during the 1970s and 1980s have revealed the highest rates in Western Europe and the US and the lowest in East Asia. Age-specific rates show a slowing increase or even a decline among premenopausal women in the US and Nordic countries, while in Eastern Europe and East Asia rates are increasing in all age groups (Kohlmeier *et al.*, 1990; Hoel *et al.*, 1992). Analyses of trend patterns imply that the most important explanations of changes may be linked to birth cohort effects (Tarone and Chu, 1992), which would reflect the influence of early life exposures (Holford *et al.*, 1991). As regards incidence of breast cancer, increasing rates have been found universally during the last three or four decades (Parkin and Nectoux, 1991). In Western countries, markedly increasing trends have most importantly been explained by birth cohort effects (Hakulinen *et al.*, 1986; Ewertz and Carstensen, 1988; Holford *et al.*, 1991). Notably, in several of these high-risk countries, the increase in breast cancer mortality has been small or absent (Ewertz and Carstensen, 1988; Holford *et al.*, 1991). We reported, on the basis of breast cancer incidence in Sweden 1958-88, a significant average annual increase by 1.3%, ranging in age groups from 0.9% to 3.0% (Persson *et al.*, 1993). These trends were explained best by birth cohort effects, with about a 3-fold increase in the incidence for women born in the 1950s relative to those born in the 1880s.

The present study aims to analyse trends in breast cancer mortality in the whole population of Swedish women, 30-89 years of age, during the period 1953-92. Efforts are made to disentangle the effects of age, period and birth cohort, and to assess the effect of a policy change in cause of death classification.

Correspondence: I Persson
Received 12 May 1994; revised 6 March 1995; accepted 28 March 1995

### Materials and methods

### Materials

Data on causes of death have been collected in Sweden on a national basis since 1749. Since 1951, data have been classified and edited according to the International Classification of Diseases, Injuries and Causes of Death (ICD). At Statistics Sweden (SCB), the cause of death statements are recorded in an annually updated causes of death register, which is known to be virtually complete. In 1971, death certificates became compulsory for all deaths, but for several years before this about 99% of the death causes were proven by certificates (Causes of Death Registry, 1971 and 1994).

New complementary rules for the coding of causes of death were instituted by Statistics Sweden (SCB) and first applied to the classification of the deaths in 1981. Part I of the death certificate concerns illnesses which the doctor judges as *causing* the death. In part II, illnesses *contributing* to the death are reported. Before 1981, a malignant tumour in part II was usually picked up and registered by the personnel at the SCB as the underlying cause of death. From 1981, however, if the cause specified in part I by itself can lead to death, the malignant tumour from part II is no longer given as underlying cause of death, unless it is judged to be an evident cause of death. We carefully tested the hypothesis that the change in the classification policy in 1981 might have led to artificially lowered mortality rates due to breast cancer being registered as cause of death in a smaller proportion of patients.

### Statistical methods

This study was based on all 51 048 breast cancer deaths in women 30-89 years of age registered during the 40-year period 1953-92 (Table I). Mortality rates (number of deaths/100 000 women) were formed by dividing the number of deaths in an age group, retrieved from the annual publications from the causes of death register, by the mean population for the same group obtained from the population register of Statistics Sweden.

The data were organised in 12 5-year age groups, from 30-34 to 85-89. Period was coded as a factor for the eight

5-year periods 1953–57, ..., 1988–92. From the age groups and periods, 19 overlapping synthetic birth cohorts were constructed. Cohort 1 consists of women born in 1863–72, cohort 2 of those born in 1868–77 etc., and women born in 1953–62 belong to cohort 19. The analyses were based on data for individual years, which implied use of $12 \times 40 = 480$ observational units. This analysis of the age–period–cohort model differs from the standard procedure, which is based on 5 yearly data (implying $12 \times 8 = 96$ observational units). The reason for use of annual data was the opportunity to include a number of simple trend analyses as essentially special cases of the age–period–cohort models. This simplifies the description of the results. A more detailed comparison of the standard approach with the present one is not possible here. However, all models were also analysed in the standard way, and the results obtained were similar.

If we assume Poisson variability of the number of deaths in each group, the variance of the log of the mortality rate is the inverse of the expected number of deaths. Thus, linear models for the log rates were fitted using weighted least-squares regression with the observed number of deaths as weights. For the present type of data, this estimation method gives virtually the same estimates as the maximum likelihood (ML) method (Clayton and Schifflers, 1987*a*). The full age–period–cohort model contains all three factors, but sub-models were also estimated, including the age–drift model. Drift is the linear effect of period and/or cohort on the logarithmic rates (Clayton and Schifflers, 1987*a*). We have 5-year coding of drift, so that it is equal within a 5-year period.

### Results

Figure 1 shows the development of age-specific and age-standardised rates (standardised using the direct method, and the mean population of women in Sweden in 1970 as standard). Overall, the age-standardised rates were remarkably stable during the observed period, with a mean of 31.5 (deaths per 100 000 and year) and a standard deviation of 1.6. The rates were somewhat higher at the beginning of the 1960s and 1970s. A decline during the later part of the 1970s is followed by a stabilisation at a level slightly above 29 from the mid-1980s.

Simple trend analyses for different age groups, assuming a constant annual percentage change in death rates over the whole period, showed significant annual 0.4–0.5% reductions for the age groups 40–54 and 75–79 years. In a similar model, allowing for a change in the level of the death rates as a result of the coding change in 1981, a significant negative effect of this change was obtained for the age groups 75–89 years. The rates were estimated to be lowered by 12%, 19%

**Figure 1** Observed breast cancer mortality rates for selected 5 year age groups and age standardised to the Swedish population of women in 1970. Log scale on vertical axis.

**Table I** Breast cancer mortality rates (per 100 000) and number of deaths in women, from all of Sweden, 1953–92

| Period | 30–34 years Rate | Deaths | 35–39 years Rate | Deaths | 40–44 years Rate | Deaths | 45–49 years Rate | Deaths | 50–54 years Rate | Deaths | 55–59 years Rate | Deaths | 60–64 years Rate | Deaths | 65–69 years Rate | Deaths | 70–74 years Rate | Deaths | 75–79 years Rate | Deaths | 80–84 years Rate | Deaths | 85–89 years Rate | Deaths |
|---|---|---|---|---|---|---|---|---|---|---|---|---|---|---|---|---|---|---|---|---|---|---|---|---|
| 1953–57 | 3.5 | 47 | 9.6 | 128 | 22.6 | 303 | 36.4 | 479 | 48.5 | 582 | 58.0 | 619 | 70.7 | 654 | 85.6 | 673 | 93.4 | 563 | 115.2 | 475 | 118.9 | 259 | 149.3 | 128 |
| 1958–62 | 2.1 | 25 | 9.4 | 126 | 20.0 | 264 | 35.2 | 468 | 49.3 | 636 | 57.7 | 673 | 73.8 | 752 | 80.0 | 683 | 103.7 | 705 | 129.4 | 599 | 153.3 | 398 | 185.4 | 187 |
| 1963–67 | 2.2 | 24 | 7.8 | 92 | 17.6 | 234 | 31.2 | 408 | 50.1 | 655 | 65.2 | 821 | 71.3 | 798 | 82.6 | 782 | 94.1 | 705 | 118.0 | 629 | 140.3 | 421 | 182.9 | 228 |
| 1968–72 | 2.6 | 30 | 8.5 | 94 | 17.2 | 204 | 31.3 | 413 | 49.7 | 642 | 66.9 | 855 | 71.1 | 862 | 79.1 | 830 | 104.8 | 883 | 130.6 | 786 | 161.8 | 579 | 194.0 | 295 |
| 1973–77 | 4.8 | 70 | 7.3 | 85 | 20.7 | 228 | 29.9 | 352 | 49.0 | 638 | 61.7 | 779 | 71.9 | 887 | 87.6 | 1000 | 102.9 | 971 | 130.1 | 899 | 161.1 | 681 | 225.8 | 437 |
| 1978–82 | 3.0 | 49 | 9.0 | 131 | 15.7 | 182 | 30.7 | 336 | 42.3 | 492 | 59.8 | 762 | 71.1 | 868 | 83.2 | 971 | 100.9 | 1046 | 116.6 | 922 | 161.6 | 805 | 210.0 | 500 |
| 1983–87 | 3.9 | 56 | 9.1 | 146 | 17.0 | 247 | 27.8 | 322 | 43.9 | 475 | 62.2 | 709 | 69.3 | 854 | 77.1 | 895 | 95.1 | 1015 | 111.2 | 985 | 134.5 | 797 | 173.2 | 511 |
| 1988–92 | 2.7 | 38 | 10.9 | 158 | 18.7 | 301 | 33.0 | 477 | 42.7 | 489 | 56.1 | 598 | 68.2 | 757 | 75.3 | 888 | 94.1 | 1008 | 110.5 | 1021 | 137.0 | 925 | 163.5 | 589 |

and 31% respectively, in the three age groups. More substantial changes in trend effects, compared with the models that did not account for the coding change, were obtained for age group 45–49 (an annual decrease of 0.8%) and for the two oldest age groups (annual increases of 0.6% and 1.2% respectively).

In models without the coding change dummy, an assumption of a constant annual change in death rate did not represent the data correctly for many age groups. This was seen when a second-order trend term was added to the basic model. Strongly significant negative parameters were obtained for the second-order term for the four oldest age groups, reflecting a decrease in death rates towards the end of the observation period in the oldest age groups. Addition of the coding dummy to the second-order trend model produced a significant positive second-order term for the age group 40–44 and significant negative second-order terms for the age groups 55–59, 70–74, 80–84 and 85–89. A significant effect of the coding dummy was only found for the oldest age group (implying a reduction by 22% as a result of the change in coding practice).

These results indicate that it is difficult to distinguish the effect of the change in coding practice from a possible non-linear trend effect for the oldest age groups. However, an effect of the coding change seems to be clear at least in the oldest age group.

Figure 2 presents graphically the age-specific mortality rates for different birth cohorts. The curves overlap closely and there is no easily discernible pattern by birth cohort present.

In the age–period–cohort modelling, drift made a significant improvement on the model containing age alone. Adding period or cohort improved the age–drift model, thus indicating non-linear period and cohort effects. The full age–period–cohort model was a further improvement on either of these submodels. Age alone explained virtually all of the variation in the logarithmic rates (99.9%). The remaining variation, 0.1%, was reduced by 11% when period was added to the age model. Cohorts produced a reduction of 14%, and period plus cohorts 19%.

The age effects obtained from the different models are very similar, and well represented by Figure 2. It is noted that the increase in mortality rates slow down from the age of 50 and during the subsequent 10–15 years of age. This corresponds to the so-called Clemmesen's hook, previously described for age-specific breast cancer incidence.

From the age–period model, mortality seems rather constant over the time periods (Figure 3). There is no marked pattern of variation and the relative risk estimates are very close to 1. For the period 1973–77, a slightly higher relative risk (5%) is noted as compared with the reference period 1953–57. The two last periods (1983–92) have significantly lower risk (6%) than the reference period, which could be explained to some extent by the change in coding practice in 1981.

Even though Figure 2 did not reveal a specific pattern, the age–cohort model manages to trace a weak downward trend starting with women born around 1888 (see Figure 4). The risk for this cohort is estimated to be 11% higher than for the reference cohort of women born around 1903, whereas women born around 1938 have an estimated risk 15% below the reference.

### Discussion

Our analyses of breast cancer mortality during the 40 year period 1953–92 in Sweden revealed quite clear-cut patterns. Breast cancer mortality was remarkably stable over the study period. Age alone explains almost all of the breast cancer mortality. A slight decrease in mortality was noted in the two latest 5-year periods, and a weak downward trend was observed with successively younger birth cohorts from about 1890.

Concerning the age–period–cohort modelling of trends, some methodological considerations are in order. In the full model, separation of the three effects age, period and cohort is impossible, owing to the fundamental identification problem in such models (Clayton and Schifflers, 1987*b*). Only non-linear period and cohort effects can be estimated, while the linear effects cannot be separated. By introducing further assumptions, the identification problem can be solved (Holford, 1991). Since a priori knowledge on which of the two variables, cohort or period, would provide the most convincing biological explanation does not exist, it was not possible

**Figure 2** Observed breast cancer mortality rates by age (midpoint of the age group) in 19 overlapping birth cohorts of women born 1863–72, 1868–77, ..., 1953–62. Log scale on vertical axis. *Note*: Since the curves are not separated, information on which curve belongs to which cohort is less interesting and therefore not given.

**Figure 3** Period effects estimated from the age–period model, reference period 1953–57, and 95% confidence intervals. Log scale on vertical axis.

**Figure 4** Cohort effects estimated from the age–cohort model, reference cohort 1898–1907, and 95% confidence intervals. Log scale on vertical axis. *Note*: The three youngest cohorts are omitted.

to specify restrictions in the full model that would have solved the identification problem. As the magnitude of the effects associated with period and cohort were small, we did not try to separate the effects by considering various assumptions. Instead, we report the effects of age, period and cohort from the respective submodels. Age–period–cohort modelling offers a considerable advantage over simple descriptive methods. With this approach, it is possible to test whether a significant improvement is obtained when period or cohort is added to a model containing age alone, and to quantify the effects. It can then be decided if the full age–period–cohort is an improvement on the age–period or the age–cohort model.

When interpreting these long-term trends, it is necessary to consider possible artefacts in the registration and coding of the data. A change in the coding practice made in 1981 could reduce the likelihood of breast cancer being registered as a cause of death. Our analyses indicate that the mortality rates could have been artificially lowered for at least age group 85–89, and probably from the age of 75 years. For these older women, other competing causes of death are important. The decrease in the mortality of post-menopausal breast cancer attributatble to the change in coding policy has been estimated by others to be about 5% (Kohlmeier *et al.*, 1990). This possibility also affects interpretation of the results from the age–period–cohort modelling. Thus, part of the lowered risk during the last two 5 year periods might be due to this change in coding practice. It is, however, difficult to judge whether an improvement in treatment, expected to benefit all age groups equally, also contributed.

Furthermore, declining autopsy rates – from some 40% in the 1970s to around 30% in the 1980s (Statistics Sweden, 1993) – might affect the number of ascertained breast cancer deaths. However, the proportion of all breast cancers found incidentally at autopsy has been low (below 1%) and has not changed since the mid-1970s (The Cancer Registry, 1980, 1983, 1989, 1993).

Observations similar to ours, i.e. a constant increase in breast cancer *incidence* and an absence of a corresponding increase in *mortality* in the recent 30 year period, have been reported from other countries. Thus, a continuous increase in invasive breast cancer incidence in successive birth cohorts, and a declining or stable mortality in younger cohorts, was found by Holford *et al.* (1991) in the US during the period 1950–84. In the study by Ewertz and Carstensen (1988), based on statistics from all of Denmark during the period 1943–82, incidence and mortality rates were increasing in parallel up to 1960. After this, the trends in incidence and mortality became different, with decreasing mortality among the oldest women. It is notable that in other countries, especially in Eastern Europe and East Asia, an increasing incidence appears to be accompanied by increasing mortality (Kohlmeier *et al.*, 1990; Hoel *et al.*, 1992). Overall, breast cancer mortality is lower in the Nordic countries than in other countries in Western and Eastern Europe (la Vecchia *et al.*, 1992). The age-adjusted rates (based on the US population in 1986) for females aged 45–84 years are 71 *vs* 88 and 76 respectively (Hoel *et al.*, 1992).

Our observations, along with those from the US and other Scandinavian countries, imply that survival after breast cancer has been improving over the last 30–40 years. In Sweden, 5 year survival in breast cancer patients has indeed become better, improving by 29% over a 19 year period (Adami *et al.*, 1986). The possibility for earlier detection is real, through increasing access to health care and awareness of health issues, and notably by the institution of mammography examinations. In Sweden, the use of mammography was infrequent in the 1970s and increased only gradually up to the late 1980s, when screening programmes in almost all of Sweden were launched.

Therefore, it seems difficult to explain the absence of mortality increase over the study period only by earlier detection. Another possibility is better survival due to surgical, radiological, adjuvant cytotoxic or hormonal treatment (Harris *et al.*, 1992*a*; Sacks and Baum, 1993). However, its impact is uncertain, since the increasing divergence between incidence and mortality has been ongoing for more than three decades. Other unknown influences on the natural course of breast cancers might be important, e.g. the occurrence of successively less aggressive tumours (Adami *et al.*, 1986; Harris *et al.*, 1992*b*) or an enhanced host defence against tumour cells. Considering the markedly varying patterns in incidence and mortality trends among countries, factors tied to influences of relatively short-term changes in lifestyle factors seem likely. With regard to the occurrence of breast cancer, nutritional factors, e.g. calorie or fat intake at an early age, have been proposed to be important aetiological factors (Trichopoulos, 1988; Harris *et al.*, 1992*b*). Whether lifestyle factors are important determinants of breast cancer survival is not known.

Our findings, however reassuring with regard to trends in breast cancer mortality, highlight urgent research issues, regarding both the aetiology and the biology of breast cancer.

**Acknowledgements**
This work was supported by grants from the Swedish Cancer Society.

### References

ADAMI H-O, MALKER B, RUTQVIST L-E, PERSSON I AND RIES L. (1986). Temporal trends in breast cancer survival in Sweden: significant improvement in 20 years. *J. Natl Cancer Inst.*, **76**, 653–659.

CLAYTON D AND SCHIFFLERS E. (1987*a*). Models for temporal variation in cancer rates. I. Age–period and age–cohort models. *Stat. Med.*, **6**, 449–467.

CLAYTON D AND SCHIFFLERS E. (1987*b*). Models for temporal variation in cancer rates. II. Age–period-cohort models. *Stat. Med.*, **6**, 469–481.

DE WAARD F AND TRICHOPOULOS D. (1988). A unifying concept of the etiology of breast cancer. *Int. J. Cancer*, **41**, 666–669.

EWERTZ M AND CARSTENSEN B. (1988). Trends in breast cancer incidence and mortality in Denmark 1943–1982. *Int. J. Cancer*, **41**, 46–51.

HAKULINEN T, ANDERSEN AA, MALKER B, PUKKALA E, SCHOU G AND TULINIUS H. (1986). Trends in cancer incidence in the Nordic countries. A collaborative study of the five Nordic Cancer Registries. *Acta Path. Microbiol. Immunol. Scand. A.*, **288** (Suppl.), 62–63.

HARRIS JR, LIPPMAN ME, VERONESI U AND WILLETT W. (1992*a*). Breast cancer, part I. *N. Engl. J. Med.*, **327**, 319–327.

HARRIS JR, LIPPMAN ME, VERONESI U AND WILLETT W. (1992*b*). Breast cancer, part III. *N. Engl. J. Med.*, **327**, 473–479.

HOEL DG, DAVIS DL, MILLER AB, SONDIK EJ AND SWERDLOW AJ. (1992). Trends in cancer mortality in 15 industrialized countries, 1969–1986. *J. Natl Cancer Inst.*, **84**, 313–320.

HOLFORD TR. (1991). Understanding the effects of age, period and cohort on incidence and mortality rates. *Ann. Rev. Public Health*, **12**, 425–457.

HOLFORD TR, ROUSCH GC & MCKAY LA. (1991). Trends in female breast cancer in Connecticut and the United States. *J. Clin. Epidemiol.*, **44**, 29–39.

KOHLMEIER L, REHM J AND HOFFMEISTER H. (1990). Lifestyle and trends in world-wide breast cancer rates. *Ann. NY Acad. Sci.*, **X**, 259–368.

LA VECCHIA C, LUCCHINI F, NEGRI E, BOYLE P, MAISONNEUVE P AND LEVI F. (1992). Trends of cancer mortality in Europe, 1955–1989. III. Breast and genital sites. *Eur. J. Cancer*, **28A**, 927–998.

PARKIN DM AND NECTOUX J. (1991). The changing incidence of breast cancer. *Rev. Endocrine-Related Cancer*, **39**, 21–27.

PERSSON I, BERGSTRÖM, R, SPARÉN P, THÖRN M AND ADAMI H-O. (1993). Trends in breast cancer incidence in Sweden 1958–1988 by time period and birth cohort. *Br. J. Cancer*, **68**, 1247–1253.

SACKS NPM AND BAUM M. (1993). Primary management of carcinoma of the breast. *Lancet*, **342**, 1402–1408.

CAUSES OF DEATH REGISTRY. (1971). *Causes of Death 1969–70*. National Central Bureau of Statistics: Stockholm.

CAUSES OF DEATH REGISTRY. (1994). *Causes of Death 1992*. National Central Bureau of Statistics: Stockholm.

TARONE RE AND CHU KC. (1992). Implications of birth cohort patterns in interpreting trends in breast cancer rates. *J. Natl Cancer Inst.*, **84**, 1402–1410.

THE CANCER REGISTRY. (1980, 1983, 1989, 1993). *Cancer Incidence in Sweden 1975, 1980, 1985 and 1990*. The Swedish National Board of Health and Welfare: Stockholm.